# P-2131. Identification of Non-*fumigatus Aspergillus* Species in Clinical Samples from Southern California

**DOI:** 10.1093/ofid/ofae631.2286

**Published:** 2025-01-29

**Authors:** Emily Rayens, Jessica Skela, Sara Y Tartof

**Affiliations:** Kaiser Permanente Southern California, Pasadena, California; Kaiser Permanente Southern California, Pasadena, California; Kaiser Permanente Southern California, Pasadena, California

## Abstract

**Background:**

*Aspergillus* is the causative agent of aspergillosis, with presentations ranging from mild skin infections to disseminated disease affecting the central nervous system. Fungal culture from the infected site is the current recommendation to confirm presence of *Aspergillus* and to speciate based on morphology. *A. fumigatus* has historically been identified as the source of most serious infections, but there is emerging concern for invasive disease caused by non-*fumigatus­* species, especially those that may carry intrinsic resistance to recommended antifungals. In this descriptive analysis, we reviewed trends in *Aspergillus* species cultured from a large clinical cohort.

**Figure 1.**
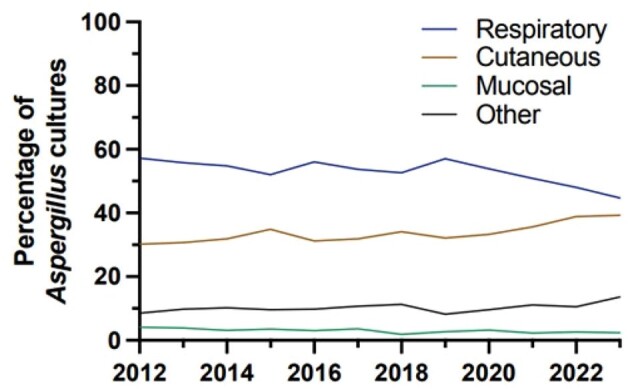
Percentage of Positive Aspergillus cultures by specimen origin type over time 2012-2023.

**Methods:**

We identified all positive fungal cultures for at least one *Aspergillus* species across Kaiser Permanente Southern California hospitals and clinics from January 2012–June 2023. Episodes were defined as a combination of any *Aspergillus* culture results conducted within 30 days of the first positive *Aspergillus* culture. Only the first episode for each patient was included in analyses if multiple episodes were identified.

**Figure 2.**
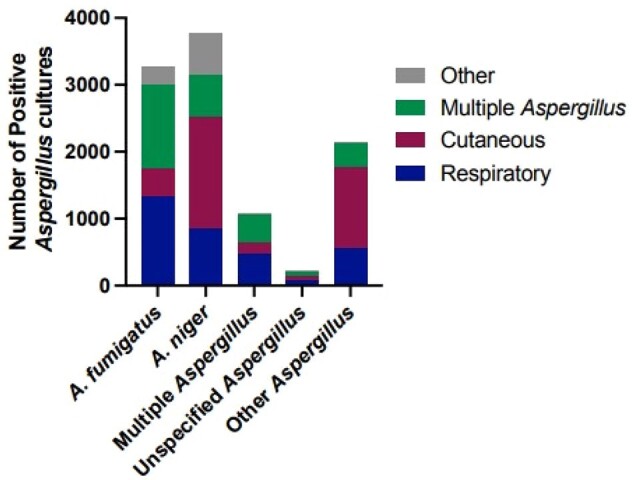
Number of positive Aspergillus cultures by species identified and specimen type.

**Results:**

10,777 patients were identified at KPSC with at least one positive *Aspergillus* culture between 2012 and 2023. Isolates were most frequently identified from samples associated with the respiratory tract and bronchoalveolar lavage fluid. However, the proportion of cutaneous cultures has increased over time and was nearly equal to the number of positive respiratory cultures in 2023 (Figure 1). *Aspergillus niger* (n=3,772) was the most frequently cultured *Aspergillus* species overall, with 44.6% of isolates originating from skin, nail, and scalp samples (Figure 2). *Aspergillus fumigatus* was identified in 40.3% of respiratory sample cultures, followed by *Aspergillus niger* at 25.7%. Finally, non-*fumigatus* specimens exhibited seasonal trends in respiratory samples compared to *A. fumigatus*, peaking in early autumn each year (Figure 3).

**Figure 3.**
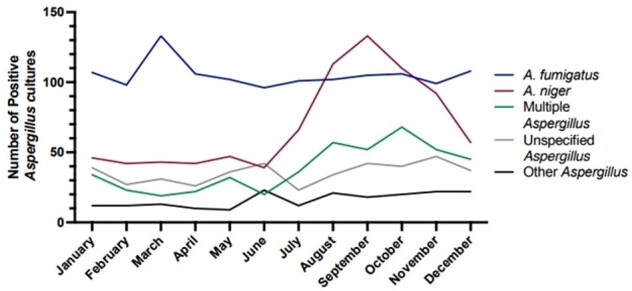
Number of positive Aspergillus cultures in respiratory samples by reported species by month.

**Conclusion:**

*Non-fumigatus* species were identified more frequently than *A. fumigatus* in culture of respiratory and non-respiratory clinical samples. Novel observations of seasonal trends and increasing predominance of non-*fumigatus* species may indicate a shift in clinical expectations and treatment options during management of aspergillosis.

**Disclosures:**

Emily Rayens, PhD, MPH, GSK: Grant/Research Support|Moderna: Grant/Research Support Sara Y. Tartof, PhD MPH, GSK: Grant/Research Support|Pfizer Inc: Grant/Research Support

